# Deep Learning Models Optimization for Gait Phase Identification from EMG Data During Exoskeleton-Assisted Walking

**DOI:** 10.3390/biomimetics10090617

**Published:** 2025-09-13

**Authors:** Roberto Soldi, Bruna Maria Vittoria Guerra, Stefania Sozzi, Leo Russo, Serena Pizzocaro, Renato Baptista, Alessandro Marco De Nunzio, Micaela Schmid, Stefano Ramat

**Affiliations:** 1 Laboratory of Bioengineering, Department of Electrical, Computer and Biomedical Engineering, University of Pavia, 27100 Pavia, Italy; roberto.soldi01@universitadipavia.it (R.S.); brunamariavittoria.guerra@unipv.it (B.M.V.G.); stefania.sozzi@unipv.it (S.S.); leo.russo01@universitadipavia.it (L.R.); spizzocaro@lunex.lu (S.P.); micaela.schmid@unipv.it (M.S.); 2 Department of Research and Development, LUNEX International University of Health, Exercise and Sports, Avenue du Parc des Sports, 50, 4671 Differdange, Luxembourg; rbaptista@lunex.lu (R.B.); adenunzio@lunex.lu (A.M.D.N.); 3 Luxembourg Health & Sport Sciences Research Institute ASBL, Avenue du Parc des Sports, 50, 4671 Differdange, Luxembourg

**Keywords:** gait analysis, sEMG, exoskeleton, deep learning, hyperparameter tuning

## Abstract

Exoskeletons are a fast-growing technology that enables multiple use-cases in clinical scenarios. They can be useful tools for the rehabilitation of patients with motor dysfunctions caused by neurological conditions, aging or trauma. Assistive exoskeletons modulate the torque exerted by the electrical motors moving their joints to allow the patients wearing them to achieve an intended movement, such as gait, correctly. Their effectiveness, therefore, requires accurate online control of such torques to complement those generated by the patient. Hereby we explored Deep Learning (DL) models to generate an online prediction of the gait phase, i.e., stance or swing, during assisted walking with a lower-limb exoskeleton based on surface electromyography (sEMG) data. We leveraged the lead of muscular activation with respect to the movement of the limbs to adjust the labeling based on joints kinematics. The cross-subject design allowed to generalize over subjects not considered for training A hyperparameter optimization algorithm was also implemented to further explore the capabilities of DL models of a reduced size. We simulated a use case scenario to assess whether online implementation of the proposed technique is feasible. We also proposed a new metric called trade-of score (*TOS*) for evaluating the cost-performance compromise of the optimized models which lead to identifying a DL model capable of classifying gait phases with an accuracy of about 95% while significantly reducing the number of parameters compared to the full architecture. Its mean computational time of less than 10 ms offers the opportunity for accurate, online exoskeleton control based on sEMG data.

## 1. Introduction

In healthy individuals, normal walking is a method of locomotion that involves the alternating use of the two legs to provide both support and propulsion [1,2]. Walking is a complex and repetitive task performed in a cycle that alternates between two main phases for each lower limb: the stance phase, when the foot is in contact with the ground, and the swing phase, when the foot is off the ground [3]. The gait cycle refers to the sequence of actions and to the temporal extent between two occurrences of the same walking event [4]. It is typically studied by considering the heel-strike as the starting event and the subsequent heel-strike of the same foot as the final event. Each cycle consists of several subphases for each leg: loading response, mid-stance, terminal stance, pre-swing, initial swing, mid-swing, and terminal swing. At heel strike, the heel makes contact with the ground initiating the loading response. At this time, the hip of the loaded leg is flexed between 20° and 30° and the knee angle is small, about 10° [2], the weight is supported by the Gluteus Maximus and the Biceps Femoris (BF), which are active together with the Rectus Femoris (RF) and the Tibialis Anterior (TA) [5]. Such leg just entered the stance phase, and the center of mass needs to be pushed forward in order to proceed walking. The contralateral lower limb, which is still touching the ground when the considered heel-strike occurs, is in pre-swing and pushes the body forward thanks to the action of the Soleus (SOL) and Triceps Surae [5,6]. During mid-stance, the loaded leg supports the entire weight of the body thanks to the activation of the vasti (Vastus Intermedius, Lateralis (VL) and Medialis). The ipsilateral hip extends, up to about −20°, the knee is fully extended and flexes to 20–30° in late stance [2]. At the end of the stance phase, several muscles are involved to reach toe-off: the hip flexor muscles (Adductor Longus, Sartorius, Iliacus, and Gracilis muscles), the BF, the TA, and the Extensor Digitorum Longus. Consequent to the toe-off, the leg enters the initial-swing phase; the TA is still active in order to maintain the ankle position and dorsiflexion of the foot for the entire mid-swing phase. The knee angle reaches its maximal flexion (50–60°) during mid-swing while the hip is flexing, and the foot moves forward preparing for the end of the swing phase. During the terminal swing Hamstrings, RF and TA are active [6], extending the knee and preparing a new heel strike.

The essential ability of walking is often compromised either by lesions affecting the central nervous system, whether in the brain (e.g., stroke) or in the spinal cord, or by neurodegenerative diseases such as Parkinson-plus syndromes and multiple sclerosis [7,8,9,10]. Gait assistance and rehabilitation can be achieved using assistive lower-limb exoskeletons [11,12,13], supportive robotic tools that contribute to the execution of the gait cycle. Nowadays most of the assistive exoskeletons rely on a time-fixed control strategy that totally or partially guides the patient to execute a predetermined gait pattern in a predefined time interval [14,15,16]. For this purpose, the controlled variable is the angle of the targeted joints: hip, knee and ankle. The control software modifies the torque of joint actuators in order to produce the predetermined joint angles trajectory in time [14].

While this control can provide a safe and effective approach to standardized gait training [17,18,19], personalized and active therapy may lead to improved outcomes compared to the time-fixed passive paradigm. Evidence suggests that active-assisted walking supports both the functional recovery of the lower limbs and the facilitation of neuroplasticity, providing patients with a dual therapeutic benefit [20]. Pursuing a personalized approach that enables effective human–robot interaction is fundamental for implementing a volitional control of the exoskeleton that is able to actively support patient motion. Online evaluation of biomechanical and bioelectric signals to predict human motion is a fast and safe strategy that grants rapid responses to the patient’s behavior. This approach enables the integration of the effort and commitment put forth by the patient into exoskeleton control, leading to more effective gait rehabilitation [21].

Han et al. [22] implemented a multi-joint continuous motion estimation leveraging regression deep learning (DL) models; they reconstructed lower limb angle trajectories from surface electromyography (EMG) signals obtaining good results for knee joint angle reconstruction (R^2^ = 0.9718, RMSE 1.2648°). In spite of the promising results, their setup was limited to walking without any robotic aid, while the gait pattern resulting from using an exoskeleton might have some important differences compared to free gait in healthy controls. In particular, while walking with an exoskeleton the sagittal range of motion of lower extremities is altered, resulting in a reduction for the hip and ankle and an increase for the knee [23,24].

More recently, Liang et al. [25] applied a convolutional transformer model to achieve a continuous prediction of the knee joint angle using sEMG. They implemented the system on data recorded while walking on a flat surface with a lower-limb exoskeleton, achieving a low RMSE (3.8°) and a fast-executing model. However, the study included only five subjects, which are too few to ensure robust results or allow generalization to new subjects.

Another approach was attempted by Morbidoni et al. [26] who performed gait phase estimation using sEMG data and DL models. They differentiated the two main phases thanks to switch-sensors under the feet that helped identify the timestamp of heel-strike and toe-off. They achieved classification accuracy up to 94%. Their work, though, did not incorporate measurements during assisted walking with an exoskeleton, thereby limiting the applicability of the results within the specific experimental scenario.

Park et al. [27] explored the classification of the main gait phases during treadmill walking with a lower-limb exoskeleton based on EMG data. The authors compared three machine learning models (decision tree, k-nearest neighbors, support vector machine) and one deep learning model showing that muscle synergy features improved classification performance compared to conventional EMG features. However, the study was conducted on a small group of healthy young subjects, with training and testing performed intra-subject only, without considering real-time implementation issues.

In our previous work [28], a similar approach has been implemented to pursue online exoskeleton control by leveraging DL models for identification of the main gait phases (stance and swing) during exoskeleton-assisted walking. Data was labelled based on kinematics transduced by IMU signals and high levels of accuracy were achieved, over 93%, in different walking scenarios (over ground and on a treadmill). In the final considerations of the work, a different possible approach arose for the labeling procedure which takes advantage of the anticipatory nature of muscular activation with respect to the kinematics of gait [29]. In particular, it has been demonstrated that heel-strike and toe-off can be predicted 100–200 ms in advance by measuring EMG activity, both in healthy individuals and in amputees [30,31].

In this work, the available physiological knowledge was leveraged to pursue a fast and accurate online identification of the main gait phases. The kinematics-based labeling was adjusted by introducing a lead according to the literature to investigate whether this could lead to an improvement in detection accuracy. Furthermore, an additional analysis of DL solutions for accurate online classification of gait phases from sEMG data was carried out, starting from the previous study [28]. Several model architectures were introduced, and an in-depth analysis of computational cost, along with a simulation of the online implementation, was conducted. Five different architectures were investigated aiming to find the best trade-off between computational effort and predictive performance. Recurrent Neural Networks are one of the best suited architectures for time series classification tasks, as they process sequential data hierarchically by “storing” information in an internal state, which builds a representation of past and current data [32]. Convolutional Neural Networks, usually applied to image processing tasks, can be well-performing architectures also in time series classification. This is made possible by Temporal Convolutional Networks that can capture long-range temporal patterns and can easily handle variable-length sequences to compute new features [33]. Finally, context-based architectures like Self-attention are worth exploring due to their ability to determine the importance of each component in an input sequence relative to another associated sequence [34].

A detailed computational time analysis was conducted on each considered architecture to determine whether the trained model could be suitable for online implementation in rehabilitation scenarios. In order to fully explore the potential of the Bi-directional Long Short-Term Memory (BiLSTM) models, we performed a hyperparameter (HP) tuning process to choose the optimal set, aiming to preserve computational resources. The most common HP tuning method in DL is grid search, which consists of exploring all the possible combinations of HP in order to find the best suiting one according to a certain metric to be maximized or minimized. Nonetheless, this method requires a long execution time when the number of hyperparameters is high and the search interval is large, due to the exponential growth in the number of possible combinations. Alternative methods have therefore been developed, incorporating strategies to explore the hyperparameter space more efficiently. Aviles et al. [35] proposed the Grey Wolf Optimization (GWO) algorithm for RNN optimization in EMG signal classification, which is inspired by the social hunting behavior of gray wolves. This method replicates the natural hierarchy and predatory demeanor exhibited in nature. In a wolf pack, there is a clear hierarchy: the alpha wolves lead, the beta wolves support them, and the delta wolves help maintain order and protect the group. The GWO mimics this structure by ranking possible solutions: the best solution is the alpha, the second-best is the beta, and the third is the delta. All other solutions (wolves) adjust their positions based on these three leaders. The algorithm simulates how wolves surround and move towards prey. These movements help explore different areas of the solution space (exploration) and refine the search around good solutions (exploitation). Random factors and gradually changing parameters guide the balance between exploring new solutions and focusing on the best ones found so far. Genetic algorithms represent another possible approach that performs a stochastic search for the most effective combination of HP by implementing a metaphor of genetic evolution applying the principle of survival of the fittest [36]. The algorithm works on a population of candidate solutions, called chromosomes, and aims to maximize a “fitness function”, which is a measure of each chromosome’s fitness in solving the problem. In our classification problem, though, this method may not be the most suitable due to the high computational time required for training neural networks or other classifiers that are part of the fitness score computation. Consequently, we decided to rely on a Bayesian approach that enables a targeted exploration of the HP space thanks to the uncertainty modelling undertaken by the method. Bayesian optimization aims to maximize a specific profit function using a Gaussian process and an activation function to guide the sampling of the next HP set.

Although gait phase identification using EMG data has been extensively investigated, the available literature lacks comprehensive studies that tackle the specific rehabilitation scenario that is dealt with here, i.e., gait phase identification from EMG data recorded while walking wearing an exoskeleton, with the aim of controlling its torques. Many studies address the task without integrating the use of an exoskeleton or they do not consider real scenario requirements like the relatively low computational power that may be available to control the device. The novelty of this study consists of exploring different DL models to classify the two main gait phases, while leveraging the physiological anticipatory activation of the muscles prior to motion initiation to correct the labeling. Moreover, a detailed investigation of the computational load of the different proposed DL models has been conducted by means of hyperparameter optimization and a dedicated trade-off score for better model selection. Finally, the online simulation of the processing pipeline from the raw EMG data up to the prediction of the model sets the path for a future real time implementation of the proposed methodology.

## 2. Materials and Methods

### 2.1. Data Source

This study relies on the database of acquisitions gathered in our previous study [28] where we proposed a sEMG-based DL methodology for gait phase identification during exoskeleton-assisted walking.

A two-session cross-sectional study has been conducted to measure lower limb muscular activity during gait with an exoskeleton (ExoAtlet, Esch-sur-Alzette, Luxembourg). The population involved the study was composed of twenty-six young and healthy subjects enrolled from the student population of LUNEX, Differdange, Luxembourg. Their anthropometric data can be summarized by its mean and standard deviation: age 23 ± 3 years, 173 ± 10 cm height, 69 ± 14 kg weight. Subjects performed walking tasks in two different conditions: Walking Overground (WO) and Walking on a Treadmill (WT).

The exoskeleton segments were adjusted according to the measured anthropometric data for each subject. The dominant leg was then equipped with a set of four pairs of electrodes in order to measure surface electromyography of four different muscles: Biceps Femoris (BF), Tibialis Anterior (TA), Vastus Lateralis (VL) and Soleus (SOL). More EMG recordings would have provided more information, but the exoskeleton is equipped with belts and straps that are tightened around the lower limbs and the waist, so it was impossible to apply EMG probes on some muscles like the Vasuts Medialis which is completely covered. Therefore, the selection was limited to BF, TA, VL, and SOL, as these muscles allow easier placement of the electrodes. In an exoskeleton that using EMG data for gait control it would be foreseeable to integrate EMG probes directly into the belts and straps used for wearing it. Finally, a Maximal Voluntary Isometric Contraction (MVIC) for each muscle was recorded using an electronic dynamometer (K-Link, K-Invent, Montpellier, France) to obtain a reference value for data normalization. IMU (Inertial Measurement Unit) probes were also positioned on the subject in order to retrieve information about gait kinematics. The IMU and sEMG probes were synchronized via software (MyoResearch 5, NORAXON, Scottsdale, AZ, USA). Inclusion and exclusion criteria and the detailed preparation of subjects are reported in our previous work [28].

At the beginning of each session, before wearing the exoskeleton, each subject warmed up by walking 10 min on a treadmill at a fast-walking speed (4 to 6 km/h). Next, a familiarization session with the exoskeleton was performed for the subjects to become used to the new sensation of limited motion induced by the exoskeleton. After the familiarization phase, the participants started one of the two planned walking sessions, lasting 60 min each. The WO session was performed on a 10-m-long and 1-m-wide straight path. Horizontal parallel bars were positioned on the side to ensure safety. The WT session was performed on a rehabilitation treadmill (Gait Trainer 3, BIODEX, Shirley, New York, NY, USA) fitted with safety lateral bars. The average walking speed was set at 1.3 km/h during both the walking sessions, which is the maximum velocity for the available exoskeleton model.

Muscular activity was recorded at 1500 Hz through a wireless sEMG system (MyoMuscle, NORAXON, Scottsdale, AZ, USA). Additionally, hip and knee flexion angle data were recorded at 100 Hz, and then oversampled at 1500 Hz, using IMU sensors (MyoMotion, NORAXON, Scottsdale, AZ, USA) placed on the subject. During the gait tasks, through the entire trial duration, sEMG and IMU data were collected for three minutes every eight minutes, resulting in seven three-minute-long acquisitions for each subject. Please refer to our previous work [28] for a more detailed explanation of the two experimental paradigms, WO and WT, used for collecting the data.

**Figure 1:**
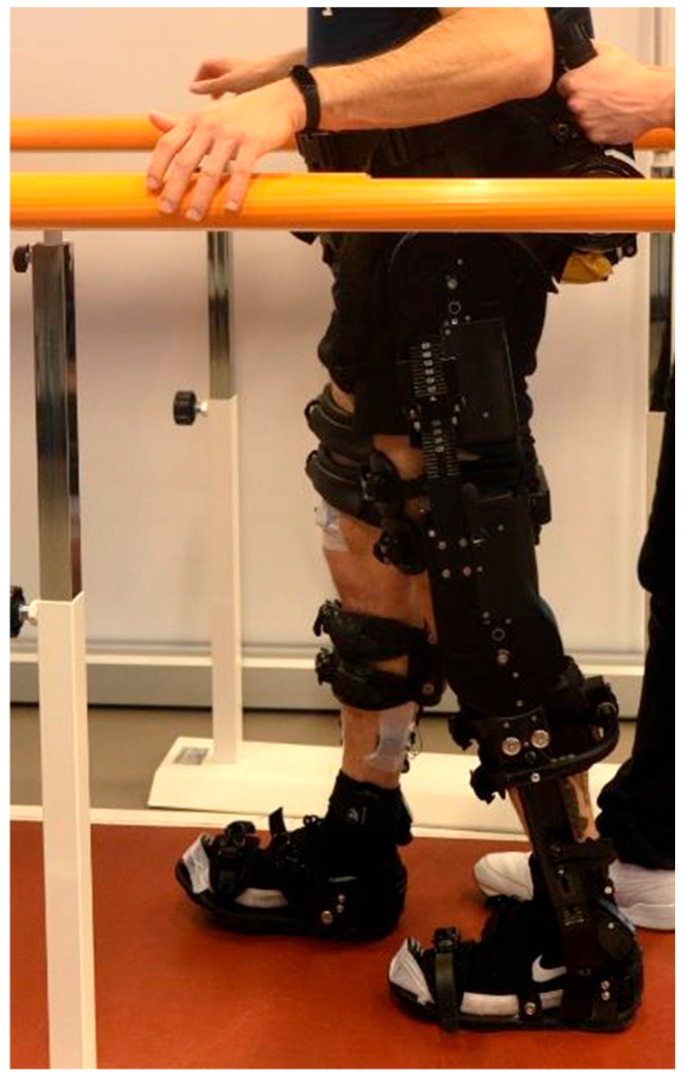
Experimental setup: a subject wearing the exoskeleton and the instrumentation used in the study (sEMG electrodes and IMUs probes) while walking overground.

Figure 1 shows a subject wearing the exoskeleton and the EMG and IMU probes during walking over ground (WO) acquisition.

### 2.2. Exoskeleton Description

The powered lower limb exoskeleton used in the study was the ExoAtlet II [37] from ExoAtlet Global SA, Luxembourg [38]. The ExoAtlet II is a CE-marked device designed to assist lower limbs motor functions of people with walking impairments of neurological or musculoskeletal nature in a rehabilitation context. The ExoAtlet II weighs around 33 kg, has a metallic structure that houses the user’s lower limbs and trunk and is equipped with four electric motors and mechanical actuators that move the hip and the knee bilaterally, providing two degrees of freedom each. The ExoAtlet II ankle joint operates in a passive mode. To ensure stability, the metallic structure is secured to the user using straps and belts. The segments are adjustable: shank and thigh length and pelvis width can be tailored to the physical characteristics of the subject’s body. The ExoAtlet II is controlled wirelessly by a tablet for the selection of different operating modes: standing up, sitting down, stepping in place, walking, and climbing upstairs and downstairs.

### 2.3. Data Preprocessing

The data preprocessing pipeline for raw sEMG data typically encompasses some standard operations like rectification and filtering in order to extract a more intelligible signal [39]. Hereby, we implemented the following pipeline: rectification, filtering, normalization, and down-sampling. We performed a rectification procedure followed by a 1st-order Savitzky–Golay filter with a window size of 39 samples [40]. Then, data normalization was performed using MVIC information gathered prior to the walking sessions. Finally, as already performed in [28], the data sampling frequency was reduced from 1500 Hz to 500 Hz by taking every third sample from the original data, since we showed that this operation does not significantly affect classification accuracy, while it reduces the computational load.

### 2.4. Data Labelling and Dataset Preparation

Since a supervised learning approach was planned for gait phase identification, a labelling procedure was necessary. We considered the two main phases of gait, stance and swing, as the two classes to be learned. In order to label the stance and swing phases, the hip (HA) and knee angles (KA) were computed by the MyoResearch software from synchronized and down-sampled (500 Hz) data gathered using IMU sensors. In the first phase of the analysis, a Hidden Markov Model (HMM) was applied to isolate and delete the quiet standing phases of the recordings, as reported in our previous work [28]. Afterwards, we implemented a threshold-based algorithm to detect heel-strike and toe-off events as the key events for discriminating between the stance and swing phases of the dominant leg. The labeling process outcome was a frame-by-frame labeling of each pre-processed sEMG data acquisition. The labelled acquisitions were then segmented into one-second-long sequences, each consisting of 500 samples, with an overlap of 90%, resulting in a 0.1 s update rate (10 Hz). Such choice should be compatible with the physiological frequency content of gait (few Hz) [41] and the timing of muscular response to external perturbations (70–100 ms) [42,43].

The label arrays were then one-hot-encoded to a binary 2 × 500 array to prepare them for training the models. The resulting dataset (WO) consisted of a total of 189,722 sequences composed of four channels, one for each muscle (BF, TA, VL, SOL), generated from the acquisitions of the 26 subjects enrolled. WT acquisitions were labeled using HA and KA data, while the HMM was not needed as there was no quiet standing phase while on the treadmill. We obtained 334,723 sequences to be considered only for testing the WO-trained model, in order to analyze whether it had the capacity to generalize to different scenarios.

**Figure 2:**
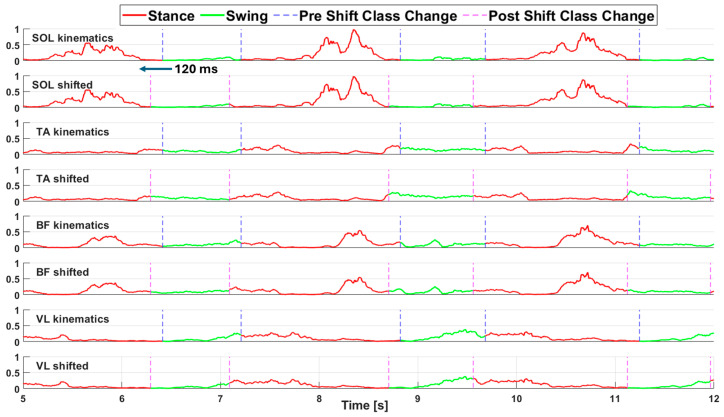
Representative portion of a sEMG acquisition (WO dataset) to be fed to the DL models. Each pair of rows shows the signal recorded from one muscle, top row with the kinematics labeling, bottom row with shifted labeling. Red trace: Stance class; Green trace: Swing class. Vertical dashed lines indicate the frame where the class changes. Blue: kinematics analysis; Magenta: shifted labeling.

We also created a second version of each dataset in which we implemented a 120 ms (60 samples) advance shift in the labeling array to leverage the anticipatory nature of muscular activity compared to kinematic activity. From this point onward the two different labeling modalities are referred to as “kinematics labeling” for the labeling strategy depending on kinematics data and “shifted labeling” for the one shifted by 120 ms. A processed portion of sEMG acquisition (before the segmentation in sequences) from the WO dataset is shown in Figure 2, both labeling modalities are reported, and a vertical dashed line highlights the frame corresponding to a change in class.

### 2.5. Deep Learning Neural Networks

We started by implementing the multi-layer BiLSTM-based neural network already proposed in our previous work [28], which we considered as a reference for its proven good performance. The model architecture, which will be referred to as “Full-BiLSTM” in the following, is composed of an input layer for sequence data (four channels, one for each muscle) followed by four BiLSTM layers set to output sequences; each one featuring 200, 100, 100, 100 hidden units, respectively. Note that the number of hidden units in a BiLSTM layer is in fact doubled to handle the bidirectionality of the layers. In order to limit overfitting, four dropout layers (with 60% dropout probability) were inserted after each BiLSTM layer. The output of the recurrent layers is fed to three cascaded fully connected layers, each one encompassing a sigmoidal activation function, having 100, 50 and 25 units, respectively. Finally, a two units fully connected layer and a Softmax layer are appended to achieve the classification task.

We then considered four other neural network architectures intending to reduce computational cost and memory usage while maintaining the best possible performance. The basic structure of each model consists of one input layer for sequence data (four channels, one for each muscle) and of the same two final layers: a two units fully connected layer and a Softmax layer for classification. Each considered model, on the other hand, encompasses its peculiar hidden layers in between. Based on previous studies suggesting that Recurrent Neural Networks (RNN) [44] are fast and effective models for EMG signal classification in different scenarios [35,45], and proving their effectiveness in gait phase identification problems [28,46], we implemented two RNN-based model architectures: one based on GRU and one on BiLSTM units. The GRU model consists of two sets of Gated Recurrent Unit layers, with 100 and 50 units, respectively, each followed by a dropout layer with a 60% probability. The BiLSTM layer has the same structure as the GRU architecture but features BiLSTM layers instead of GRU ones, implying that the number of units is doubled due to the bidirectionality in the BiLSTM layers. The choice of the hyperparameter set for recurrent networks is not a trivial one; some studies suggest setting the number of hidden units to a power of 2 [47,48,49], while others suggest choosing a multiple of 5 ranging from 10 to 150 [28,35,50,51]. However, a common approach is to set the number of units such that each subsequent layer has fewer units, often half as many as the previous one, thereby mitigating the risk of overfitting [44]. All BiLSTM and GRU layers in the models were configured to output sequences.

Then we implemented a multi-head self-attention model derived from the one presented by Xuemei et al. who leveraged attention-based neural networks to classify emotions from physiological signals [52]. The core block of the model we implemented is a 16-head self-attention layer with 256 key, query and value channels, positioned is in between two normalization layers to normalize each sequence across channels. Before and after the self-attention block, we inserted one fully connected layer with 128 units followed by a ReLu layer that introduces nonlinearity to the model and helps prevent the vanishing gradient phenomenon, and a final dropout layer with 40% probability to avoid overfitting.

Finally, the performance of a Residual Temporal Convolutional Network (Res-TCN) was also explored as it is an architecture that has already proven successful on EMG processing [53]. It is composed of three subsequent residual blocks that allow a time-dilated convolution over the layers. Each residual block is composed of a 1D Convolutional layer, a layer-normalization layer, a spatial dropout layer (50% probability), another 1D convolutional layer, another layer-normalization layer and finally a Rectified Liner Unit (ReLu) layer. The residual block structure implies a parallel data branch that skips the convolutional operations and is summed to their output feature maps. In the first skip-branch, a 1D convolutional layer is included. The optimal hyperparameter configuration of the Res-TCN model was determined by computing its Receptive Field (RF) to precisely match the temporal extent of the training sequences (500 samples), thereby ensuring full contextual coverage during training. The receptive field computation depends on four substantial parameters: number of residual blocks (*N*), number of convolutional layers for each block (*c*), dilation basis (*b*) and convolutional filter length (*k*) [54,55,56].

The computation is performed as in Equation (1)

(1) $$Rf=1+c\left(b^{N}-1\right)\left(k-1\right)$$

Reducing the possible combinations of parameters by setting the number of layers per block (*c*) to 2, the dilation basis (*b*) to 2 and the number of blocks (*N*) to 3 limited the number of learnable parameters too.

The final computation is *Rf* = 1 + 2(2^3^ − 1)(*k* − 1) and we found that the closest we could get to 500 was by setting the filter dimension (*k*) to 37, producing a receptive field of 501 samples. The number of convolutional filters is set to 64 for each 1D convolutional layer.

The training process was executed in MATLAB 2024b (The MathWorks, Inc., Natick, MA, United States) using the GPU execution environment with a NVIDIA GeForce RTX 4090 (NVIDIA Corporation, Santa Clara, CA, USA). The training options were established by running preliminary training tests and the final setup was as follows: Adam optimizer, up to 60 training epochs, initial learning rate of 0.0004, piece-wise learning rate reduction every two epochs with a drop factor of 0.9, batch size of 128, validation patience of 10 and binary cross-entropy as loss function. To ensure the reproducibility of the experiments, all sources of randomness were initialized so that, in every run, the learning process would face the same sequence of random numbers. Three different seed setups were evaluated in the experiments; the results section reports the best outcome of the three. Table 1 serves as a summary of the proposed model architectures (See Supplementary Materials for a schematic illustration of the models’ architectures Figures S1–S5).

### 2.6. Hyperparameters Optimization

We implemented a hyperparameter optimization process involving the number of hidden units in BILSTM and fully connected layers in order to reduce the number of free parameters of the previous model proposed by Guerra et al. [28]. The most straightforward method to tackle hyperparameter optimization is grid search. This method employs a brute force approach, attempting every possible combination of parameters within a specific domain to identify the optimal combination [57]. It is evident that this type of approach results in a combinatorially increasing number of trials to execute, thereby consuming significant time and computational resources. This prompted us to implement a more sophisticated method that significantly reduces the number of trials needed. We utilized Bayesian optimization in order to explore the HP space in a more efficient manner. This method derives from the well-known Bayes’ Theorem [58] and implements Gaussian processes to estimate the posterior probability distribution of a certain unknown function. In this case the unknown function is the model accuracy depending on the chosen hyperparameters. The following step of Bayesian optimization is the computation of a so-called “acquisition function” that serves as a surrogate function of the unknown initial function. This second function models the variance of the Gaussian process output defining the goodness of each point at describing the extracted posterior distribution. Maximizing the acquisition function ensures the maximization of the original function guiding the sampling of the HP space [59,60]. In this work we utilized the MATLAB built-in Bayesian optimization algorithm that implements a Gaussian regressor with ARD Matern 5/2 Kernel [61] and the Expected Improvement Plus acquisition function [62]. The optimization process stop criterion was set to 30 trials in order to limit the combinations explored and the computational time required by the experiment.

### 2.7. Cost-Performance Trade-Off Analysis

In this other section we present a custom metric that summarizes the models’ characteristics in order to help choose between similarly performing models. The proposed metric combines two measures, Accuracy delta and Parameters variation, computed with respect to the model selected for optimization, which serves as the reference. The Accuracy delta (*Ad*) is computed as the difference in accuracy between the candidate model (*Ac*) and the reference one (*Ar*), as shown in Equation (2)

(2) $$Ad\left[\%\right]=Ac-Ar$$

The *Ad* metric is considered a “profit” if it is positive and a “cost” if it is negative. The main goal is always to ensure the best model performance, so a better performing model must always be considered. The Parameters variation (*Pv*) metric, instead, is computed as the change in the number of learnable parameters in terms of percentage between the candidate model and the reference one, as shown in Equation (3)

(3) $$Pv\left[\%\right]=100\;\frac{Pc-Pr}{Pr}$$

where *Pr* is the number of learnable parameters of the reference model, whereas *Pc* is the number of learnable parameters of the candidate model. This metric is considered a “profit” if it is negative and a “cost” if it is positive, expressing the intent of reducing model complexity and computational time. Indeed, reducing the number of learnable parameters lowers model complexity and facilitates the computational learning process [63,64,65].

The two metrics are then combined in an overall score, the Trade-Off Score (*TOS*), that balances the loss in performances with the reduction in model complexity.

We chose to set a lower threshold of −1% for *Ad* to limit the allowed loss of performance in favor of reducing the number of parameters. Any model scoring below the accuracy threshold is excluded in advance.

In order to obtain a balanced score between the two metrics, though, both the Parameters variation and the Accuracy delta need to lay in the same range. This led us to focus on finding a way to compress the Parameters variation metric within the range of [−1, 1]. Therefore, a logistic transformation is applied to *Pv* in Equation (4).

(4) $$Pvc=\frac{2L}{1+e^{\alpha Pv}}-L$$

*Pvc* stands for parameter variation cost which is the output of the logistic transformation of *Pv*. The hyperparameter *L* is set to 1 to define the score’s boundaries within the specified limits. The α parameter, set to 0.05, determines the slope of the sigmoidal curve.

Following this computation an increase in the number of parameters produces a negative score, while its reduction produces a positive score.

Therefore, the final Trade-Off Score metric is computed as in Equation (5)

(5) $$TOS=\frac{{(A}_{d}+P_{vc})}{2L}=\frac{\left(A_{c}-A_{r}\right)+\left(\frac{2L}{1+e^{k(100\cdot \frac{P_{c}-P_{r}}{P_{r}})}}-L\right)}{2L}$$

**Figure 3:**
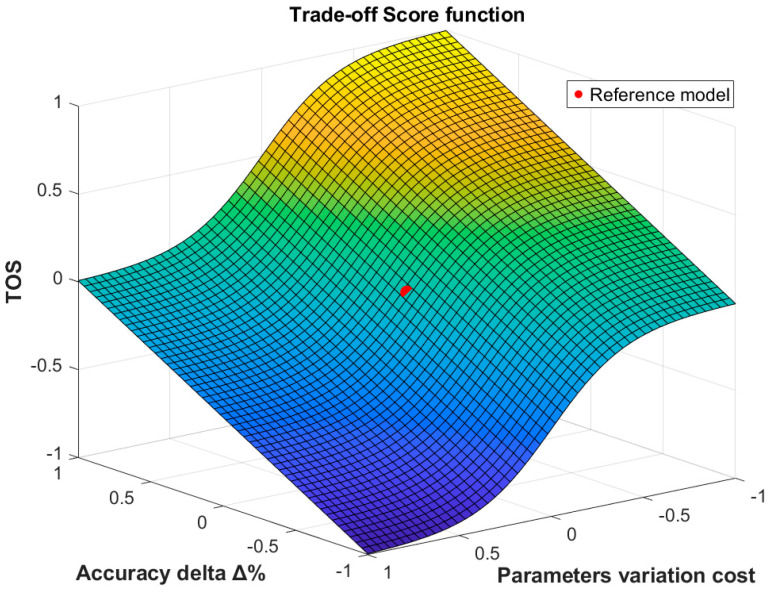
Three-dimensional surface plot of the *TOS* as a function of Accuracy delta and Parameters variation cost. Red dot: *TOS* of the reference model, i.e., 0. Color span: blue to yellow according to *TOS* value, from −1 to 1 respectively.

Normalization is applied to set *TOS* boundaries between −1 and +1. The Trade-Off Score (*TOS*) summarizes whether a model is a better compromise in terms of performance and model complexity in comparison to the reference one. *TOS* serves as a profit metric, with increasing values reflecting improved trade-off; it is equal to 0 if a model is compared to itself. The resulting trade-off metric behavior as a function of *Ad* and *Pv* is shown in Figure 3.

### 2.8. Online Implementation Simulation

The computational cost of a neural network model directly impacts the inference time, thereby introducing a delay in data processing. We simulated a possible online scenario to consider all the processing steps leading from the raw data to the model output, measuring the time needed for these computations. As explained in Data processing 2.3, the sEMG preprocessing implied rectification, filtering, normalization and down-sampling, followed by the construction of sequences. We therefore implemented an iterative simulation incorporating all these steps into a system with lower computation capabilities than the one used for training [29]. The computer used for online implementation features an Intel^®^ Core™ i7-7700 CPU @ 3.60 GHz and 32 GB of RAM.

**Figure 4:**
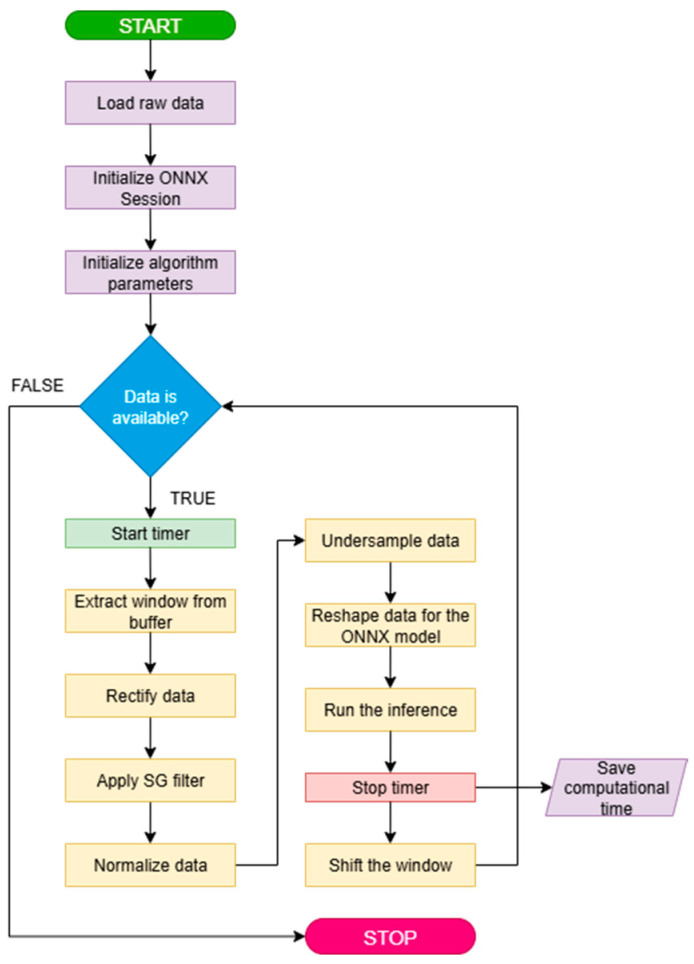
Flowchart of the online simulation setup. Purple blocks: initialization of the environment. Blue diamond: while loop control. Yellow blocks: data processing steps. Green: timer is started at each iteration.

The code for online simulations was written in Python 3.13 and features the capability of reading raw data from a “.mat” file and the ONNX (Open Neural Network Exchange [66]) file of the MATLAB exported model. The simulation of a real scenario consisted of iteratively reading a 1500-samples-long window (i.e., 1-s sequence) from the data buffer, applying the described preprocessing, down-sampling the result to 500 Hz, and then feeding it to the model to obtain a prediction. Every iteration the window is shifted by 150 samples (0.1 s). The processing time is measured through a built-in timer, and the simulation is conducted using the Intel i7 CPU mentioned above for the prediction until all the buffered data is processed. The simulation can be resumed as in the pseudo-code flowchart reported in Figure 4.

### 2.9. Experiments

#### 2.9.1. First Analysis

The first analysis was a cross-validation procedure over the original WO dataset with kinematics labeling in order to achieve the first glimpse of each model’s performance. Two different approaches were utilized for this purpose: 10-fold cross validation and Leave-One-Subject-Out (LOSO) cross validation.

#### 2.9.2. Second Analysis

The second analysis aimed to reproduce the experimental conditions used by Guerra et al. [28]. A cross-subject training process was undertaken for each model using the same 23-3 train-test division of subjects used in the previous study on the WO kinematics labeling dataset. The same train-test division of subjects was performed on the shifted labeling dataset, and the models’ performances were analyzed also on these data. Additionally, we evaluated the trained models over the WT dataset (considering both labeling approaches) to explore their generalization capabilities.

#### 2.9.3. Third Analysis

The third analysis involved an HP tuning procedure to reduce the complexity of the Full-BiLSTM model, which, as the reference model, demonstrated the highest performance but also featured a large number of parameters. We removed one BiLSTM layer and one fully connected layer from the full architecture to evaluate whether reducing the model’s depth would impact performance and computational time. The resulting new parametric architecture features one BiLSTM layer with 2*P* hidden units followed by two BiLSTM layers with *P* hidden units each. Each BiLSTM layer’s output is fed to a dropout layer with 60% probability. The fully connected layers are now three. The first layer consists of 2*M* hidden units, the second of *M* units, and the last has two hidden units to match the output dimension. The first two fully connected layers are followed by one sigmoid activation function layer and one dropout layer (60% probability) each. The last fully connected layer is fed into a Softmax layer to reconstruct the probability scores for each class.

This new architecture (see Figure S6 in Supplementary Materials) encompasses two main hyperparameters, *M* and *P*, which are the object of hyperparameter optimization. The parameter space was defined by the boundaries of *M* and *P* which are set to [15, 50] and [20, 100], respectively, in order to limit the size of the model and the computational load. The upper limits of the search space for HP were chosen so that the maximum number of neurons (2*M* and *2P*) would match those used in the largest layers of the Full-BiLSTM model. The stop criterion of the algorithm is set to 30 trials and the metric to optimize is the accuracy on the validation set. The optimization process was carried out on both the original “kinematics labeling” dataset and the “shifted labeling” version, using the 23-3 cross-subject approach, in order to compare the results with those reported in the previous study [28].

The final step involved selecting the best trade-off model, using the *TOS* metric we proposed, identifying the model that strikes the best compromise among those produced by the optimization process. We selected the best model for both the kinematics labeling and shifted labeling datasets.

#### 2.9.4. Computational Cost Analysis

A final analysis was conducted in order to measure the computational time of the Full-BiLSTM model, the Pruned-BiLSTM models and the new proposed ones. All the measurements were recorded on the models trained with the cross-subject (23 train-3 test division) method.

## 3. Results

### 3.1. First Analysis: Cross-Validation Approach

In the first experiment, five neural network model architectures were trained and tested on the entire WO dataset using a cross-validation (CV) approach with both 10-fold and LOSO approaches. A summary of the results of the trained networks on the training set is reported in Table 2.

The overall results highlight higher performances for each model in the 10-fold cross-validation training than the LOSO one. The two better performing models in this phase are the Small-BiLSTM and Res-TCN models. The highest accuracy in 10-fold CV is reached by Res-TCN model, scoring 89.60% accuracy. On the contrary, in LOSO-CV, the highest accuracy is reached by Small-BiLSTM model with an accuracy of 86.03%.

### 3.2. Second Analysis: Cross-Subject Approach

In the second experiment we reproduced the experimental conditions reported in [28]: we trained the five models over the 23 subjects and tested them with the remaining 3, yet here we considered the two datasets, i.e., with and without the labeling adjustments. Finally, we also tested the trained models with the WT dataset in the two labeling modalities over the same 3 subjects as WO. The Cross-subject results for WO dataset are reported in Table 3, while WT results are reported in Table 4.

The best performance is achieved by the Full-BiLSTM model with both dataset labeling methods, unveiling increased performance when the labeling is shifted in advance following the anticipatory nature of muscular activation.

The models trained on the WO dataset exhibit good performance when tested on the WT dataset, with only a small degradation in accuracy across all models.

### 3.3. Third Analysis: HP Optimization Approach

After observing the very promising performance of the Full-BiLSTM model a, we conducted a more detailed analysis of the impact of the hyperparameters on the model’s performance. The outcome of the hyperparameter optimization process is a total of 30 trained models featuring different sets of hyperparameters sampled in the HP space according to the Bayesian optimization algorithm. From this point on, for convenience, the models obtained from the HP optimization process are called “Pruned BiLSTM”. In Table 5, we report the final selected models among the 30 trained during the optimization process to be compared with the “Full-BiLSTM” model, choosing both the best performing one and the one that maximized the *TOS* metric.

We chose to report the model from which we started the optimization process, the outcome of the optimization and the trade-off analysis. The Full-BiLSTM was used as reference to compute the *TOS*; therefore, its score is equal to zero due to the nature of the score itself and the HP set cannot be directly reported due to the different layer organization. For the kinematics labeling dataset, the best Pruned-BiLSTM after optimization features 50 and 100 as *M* and *P* parameters, respectively, while the best one according to *TOS* is the one with 15 and 53 as HP set. For the second dataset, the one with shifted labels, the best Pruned-BiLSTM after optimization is also the best one according to the *TOS* metric. It features [27 57] as *M* and *P* hyperparameters, respectively, which is far from the boundary limits, suggesting the chosen HP space boundaries are appropriate for the problem at hand.

**Figure 5:**
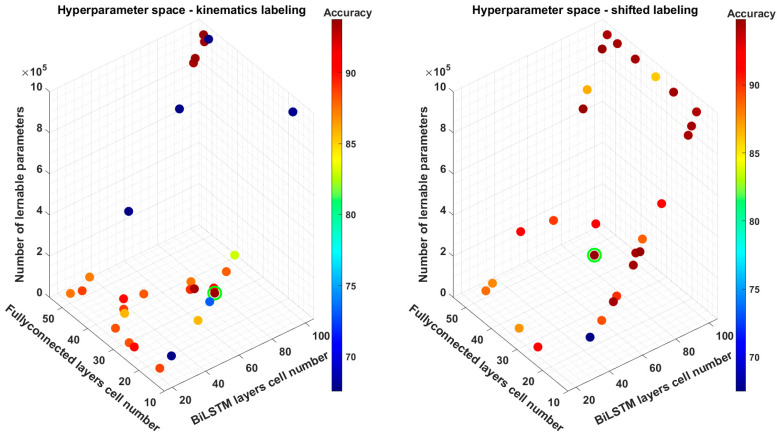
Three-dimensional Scatter plots of the accuracy of the models trained based on the Bayesian optimization procedure of *M* and P. The X axis represents P, and the Y axis represents *M*. Z axis: number of learnable parameters in the model based on the chosen *M* and P. Dots: color-coded accuracy achieved by each model. Left panel: models trained over the kinematics labeling dataset, Right panel: models trained over the shifted labeling dataset. In each panel, the green circle shows the best trade-off model according to *TOS*.

We also analyzed how the hyperparameters set influenced the performance of the model over the 30 tested combinations. In Figure 5, we report a 3D scatter plot showing the accuracy of the tested models depending on the two hyperparameters and the total number of learnable parameters. The sparsity of the graph directly shows the sampling strategy of Bayesian optimization that enables the exploration of the HP space while efficiently reducing the number of combinations evaluated.

### 3.4. Computational Cost Analysis: Online Simulation

The computational analysis was conducted for all the models trained in the second experiment and the ones selected for the third experiment. Table 6 summarizes the computational time required to process one 1500 samples window of raw data and obtain a prediction for each considered model. We selected the best Pruned-BiLSTM model for each dataset according to the *TOS* metric to be tested for this analysis. They are referred to as “Pruned BiLSTM kinematics” and “Pruned BiLSTM shifted”. We decided to test for computational time the models trained on the shifted labels dataset only because the computational time depends on the architecture and should not be affected from a different training set. However, we also analyzed the computational time of the Pruned BiLSTM model trained on the kinematics labels dataset, since as a result of HP optimization the final HP set differs from that of the model trained on the shifted labels dataset.

**Figure 6:**
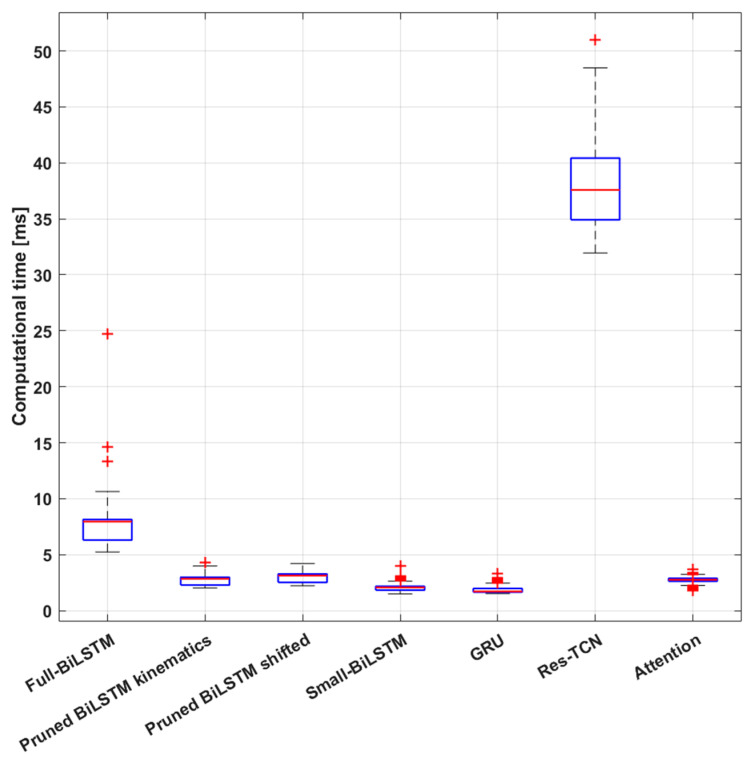
Online simulation results: the boxplots present the distribution of computational times for each model. Red crosses represent outliers (values more than 1.5 times the interquartile range away from the bottom or top of the box).

Table 6 reports the computational time (mean ± standard deviation) for each trained model, together with the maximum recorded computational time in order to understand whether it is compatible with an online implementation of the entire processing pipeline for controlling the torques of an exoskeleton based on the recorded EMG signals. Finally, memory usage was reported to provide more insight into the potential for embedded implementation of the models. Figure 6 shows more details about the online simulation. The boxplots allow the comparison of the distributions of computational time per window for each model.

## 4. Discussion

This paper provides a more detailed analysis of the implementation of DL models for online gait phase identification from sEMG data. All models are trained with the same dataset, aiming to find a well-performing model that is also suited for online implementation. The ultimate goal is to use the deep neural network model for predicting gait phases to control the actuators of an exoskeleton for assisted walking.

We exploited a pre-existing database of acquisitions collected for the previous study by Guerra et al. [28]. The database is composed of EMG data gathered from 26 young healthy subjects while walking wearing an ExoAtlet II exoskeleton. The EMG signals came from four fundamental muscles of the dominant leg: Biceps Femoris (BF), Tibialis Anterior (TA), Vastus Lateralis (VL) and Soleus (SOL). The database of acquisitions comprises two datasets: WO and WT. The first one includes acquisitions while the subjects were walking on a flat surface (WO), while the second one was recorded while walking on a treadmill at constant speed (WT). All the proposed DL models were trained with the WO dataset using different validation approaches.

The main finding of our investigation addresses the strong effects of a different labeling approach for this task. As already mentioned, muscular activation anticipates the kinematics of the gait by at least 120 ms both in healthy subjects [30] and amputees [31]. We took advantage of this physiological behavior in order to predict in advance the effects of muscular activity over gait phase initiation. This means that all the labels have a time lead of 120 ms, offering enough time to produce the commands needed to control the exoskeleton. The results we obtained when training and testing the models with the shifted-labels dataset are much more promising for the Full-BiLSTM model which reaches an accuracy of 95.04% and a F-score of 96.35% for stance and 92.30% for swing. This underlines the capabilities of a more complex model to better understand a common pattern in data which has a physiological explanation. Nonetheless, the other proposed models’ performance does not differ much when trained on the shifted labels dataset compared to when trained on the kinematics labels dataset.

The second outcome is that, as expected, the overall classification performance improves when data from the same person are included in both the training and test sets. The result is coherent with previous publications that obtained similar outcomes. Di Nardo et al. [67] implemented an intra-subject methodology for gait-event prediction using sEMG data obtaining higher performance than inter-subject approaches. Morbidoni et al. [26] reached the same conclusion while performing gait phase detection using sEMG. They obtained higher accuracy when classifying subjects included in the training set than those excluded from it, which is a typical behavior of machine learning models prone to overfitting on seen subjects. Non-cross-subject approaches, where training and test data come from the same individuals, often overestimate performance and fail to generalize correctly. In contrast, cross-subject validation separates training and test subjects, forcing the model to learn patterns that generalize well across individuals and providing a more realistic measure of performance [68]. Our results showed that the Res-TCN model is the best performing when all subjects are included in the training set, in 10-fold CV experiment, reaching an average accuracy of 89.60%, an average F-score of 92.28% for stance and 84.10% for swing. On the other hand, the small BiLSTM was the best model capable of generalizing to new subjects, in LOSO-CV, reaching an average accuracy of 86.03%, an average F-score of 89.54% for stance and 78.62% for swing. The main difference between models is that the BiLSTM ones can extract more information from data thanks to the bidirectional evaluation of the sequences both while training and testing.

A third outcome of our analysis concerns the generalization capabilities of most of the implemented models across different scenarios. We tested the models trained in the second experiment (WO dataset, 23-3 cross-subject approach) with the data of the same subjects acquired in the other walking modality (WT). The performances are comparable between the two datasets, with only a limited degradation in performance when testing WT data, never losing more than 2–3% in accuracy.

The hyperparameters optimization allowed us to explore the impact of the number of BiLSTM and of fully connected units on the model performance to try finding a trade-off between accuracy and model complexity. The model considered in this phase was the Full-BiLSTM because of its high number of learnable parameters, and their impact on computational time. We implemented a Bayesian optimization process to explore the hyperparameter space more efficiently by limiting the number of trials to 30, rather than exploring all possible combinations. The lower and upper boundaries of the hyperparameter space were set to [15, 50] and [20, 100] for the number of hidden units in the fully connected layer and in the BiLSTM layer, respectively. Among all the tested models we identified the optimal tradeoffs based on to the proposed *TOS*, which considered the Full-BiLSTM as reference. In the shifted-labels dataset we witnessed a limited reduction in the accuracy of 0.21% from 95.04% to 94.83% while significantly reducing the number of learnable parameters from 1.2 million to 325,000 (325 K in the following), i.e., 73% fewer parameters. On the other hand, for the kinematics labels dataset, reducing the number of parameters led to a slight improvement in accuracy of 0.03%. In this phase, the *TOS* played a crucial role in determining the best trade-off model. As shown in Table 5, the best performing model obtained from HP optimization scores 93.78% in accuracy, encompassing 994 K parameters while the one chosen from *TOS*, with only a small loss (−0.22%) in accuracy, needed just 278 K parameters. We also performed a sensitivity analysis of *TOS* hyperparameter *α*, which was set to 0.05 in our computations. We tested four values of *α*: 0.001, 0.005, 0.01, 0.05. For both the labeling methods the minimum value of the parameter α needed to obtain the reported results was 0.01. On the contrary, lower values led to the selection of either the best-performing network after HP tuning for the kinematics labels dataset, or the Full-BiLSTM model for the shifted-labels dataset. This result is consistent with the interpretation of the *α* parameter as the slope of the sigmoid curve. Higher values of α assign a higher weight to parameters variation, assuring a more efficient complexity reduction. This metric has been introduced since a validated trade-off score was not found in the literature. The most widely used selection strategies only rely on choosing a target accuracy or setting an upper bound to model complexity [69,70,71]. Min et al. [72] adapted the AIC (Akaike’s information criterion) and BIC (Bayesian information criterion) metrics to neural networks to investigate model selection in time series forecasting, yet these metrics rely on many hyperparameters that complicate their use. On the contrary, the proposed *TOS* metric combines the number of model parameters and the accuracy of the model into a single score that enables us to find the best trade-off compared to the reference model. Further studies could contribute to the validation of the robustness of the proposed metric.

Finally, the last result we want to discuss is the online simulation outcome. We noticed a direct impact of the number of learnable parameters on the models’ inference time. This could seem a trivial result; however, it aligns well with real-world applications of online deep learning implementation [73]. Our simulation process showed that all the models required less than 40 ms on average for the entire inference process, setting an upper bound to the online applications of 25 Hz. Upon closer examination of the results, we observe that the inference time can occasionally exceed expectations. Therefore, we must also consider the maximum inference time as the frequency-limiting value. From this point of view only the Res-TCN model exceeds the boundary, taking more than 40 ms several times during the simulation. Thus, if the online application needs to run at more than 25 Hz, the Res-TCN models need to be discarded from the candidate models. On the other hand, performance must be considered too. Excluding the Res-TCN model, the other models take less than 10 ms on average for the inference, setting a higher upper bound of 100 Hz. In this scenario we can directly compare the models in terms of performance and memory usage. The best performing model is the Full-BiLSTM one when dealing with the shifted-labels dataset. This model takes about 4.5 MB of memory just for the parameters’ values. The trade-off is represented by the Pruned-BiLSTM, which occupies much less memory, 1.2 MB only, while maintaining almost the same performance. Moreover, the processing of one window of data using this model takes on average 3 ms versus the 7.4 ms of the full model, rising the potential operating frequency to over 300 Hz. Finally, we also need to consider that we are currently using a sliding window with a step of 0.1 s that sets an upper limit of 10 Hz to the refresh frequency. By reducing this overlap, the process could be sped up to match the model’s speed. This process strongly depends on the speed at which data is transferred to the inference system and the hardware resources it runs on. However, from a physiological standpoint, a contextualized analysis is required to fully understand what operating frequency would be compatible with the needs of the biological system. Therefore, we need to compare the speed achieved during the simulation with the refresh rate of the motor commands coming from the central nervous system. Evidence has been found demonstrating the presence of Central Pattern Generators (CPGs), i.e., biological neural networks capable of producing rhythmic movements for locomotion at a cadence frequency [74,75] in the spinal cord. The frequency content of gait in healthy subjects usually never exceeds 15 Hz [41] implying an available time window of almost 70 ms to generate a motor command as a result of the DL model prediction. On the other hand, reflex responses to external perturbations that could lead to a fall are significantly faster when measured in the primary sensory cortex, approximately after 40 ms [42,43], and lead to a muscular activation in about 70 to 100 ms [43]. As a result of these considerations, the online control system may not need to operate at an exceedingly high frequency; 20 Hz should be sufficient to ensure robustness against gait perturbations, as well as occasional system slowdowns.

## 5. Conclusions and Future Perspectives

In conclusion, this study proposes a methodology for the online implementation of a DL-based gait phase identification algorithm for lower-limb exoskeleton control, leveraging the capability of predicting heel-strike and toe-off in advance through the analysis of sEMG signals. Exoskeletons should be conceived as assistive walking tools for the rehabilitation of gait dysfunctions, with control strategies tailored to the specific capabilities of each patient undergoing therapy. This implies that the exoskeleton control algorithm should be able to adapt online its commands to the joint motors to match the effort of the patient. To address this need, an analysis of deep learning models was conducted, examining their performance in relation to dataset construction, labeling methodology, and model architecture, and computational load.

The main result confirms the efficacy of RNN models for signal classification [44,45,46], highlighting the performance of Long-Short Term Memory architectures [28,40]. We also confirmed the importance of considering a cross-subject design for addressing the models’ generalization abilities in multiple-subject training and testing scenarios in the correct manner [26,67]. The new contribution this work provides to the topic is the methodological approach for gait-phase identification from sEMG data utilizing DL models. We took advantage of physiological knowledge about muscular activity [30,31] in order to improve model performance by shifting the labeling of gait phases to reflect muscle activations instead of kinematic events. We also considered the needs of an online exoskeleton control system and followed an approach that preserves computational resources. The resulting “Pruned-BiLSTM” model with shifted labeling approach, achieves high accuracy, almost 95%, while significantly reducing computational requirements compared to the initial “Full-BiLSTM” model.

From a critical perspective, this study may have some limitations. First of all, we only considered one-second-long sequences of data, overseeing that sequence length may impact on the models’ performance. In particular, the Res-TCN model, having a receptive field was fixed to sequence length, may directly benefit from shorter sequences as it would lower the number of learnable parameters and processing time. Secondly, we did not measure the advance activation of the muscles with respect to kinematics on each specific subject, but relied instead on literature data [30,31]. Future developments of this work should adjust the acquisition protocol to study each subject’s anticipatory muscular activation to correctly define the labeling. This could be achieved by recording synchronized kinematics and sEMG together with foot switches or insoles for heel-strike and toe-off identification, to measure the real lead in the activation of each muscle. Furthermore, we only considered predicting the next macroscopic phase of gait to generate a standardized control command to be sent to the actuators, yet gait is an ensemble of many sub-phases, i.e., loading response, mid-stance, terminal stance, pre-swing, initial swing, mid-swing and terminal swing. Predicting each of these phases would certainly be more effective for online exoskeleton control. The labeling could likely be based on defining each subphase as a fixed percentage of the corresponding main phase duration. Another limitation of the study concerns the enrolled subjects, who were exclusively young and healthy. This restricts the immediate applicability of our findings; however, we have no reason to believe that gait phases would not be correctly identified in patient gait. Future tests will certainly include real patients, ensuring that age and pathological features, like a different muscular activation, are considered when training the DL models.

## Figures and Tables

**Table 1:** Summary of the neural network models architectures. The number in parentheses is the figure number in the Supplementary Materials.

Model	Architecture	Number of Layers	Number of Learnable Parameters
Full-BiLSTM (S1)	Multi-layer BiLSTM and fully connected	19	1.2 M
Small-BiLSTM (S2)	Double BiLSTM	7	184.6 k
GRU (S3)	Double GRU	7	54.2 k
Res-TCN (S4)	Residual Time dilated Convolutional Network	28	768.8 k
Attention (S5)	Multi-headed self-attention	13	149.8 k

**Table 2:** Performance metrics of the 10-fold and LOSO cross-validation on the kinematics labeling dataset. The highest values for each column are in bold. The values are reported as the mean performance score over the total number of test sets generated during the training.

Model	10-Fold Cross-Validation			LOSO Cross-Validation		
	Accuracy	F score Stance	F Score Swing	Accuracy	F Score Stance	F Score Swing
Full-BiLSTM	87.68 ± 8.33	90.53 ± 6.64	82.29 ± 11.17	83.70 ± 9.79	87.59 ± 8.76	76.83 + 11.64
Small-BiLSTM	88.94 ± 1.22	91.81 ± 0.91	82.95 ± 1.92	**86.03 ± 6.19**	**89.54 ± 4.99**	**78.62 ± 8.61**
GRU	85.15 ± 1.54	89.00 ± 1.06	77.11 ± 2.98	83.26 ± 5.59	87.43 ± 4.97	74.32 ± 7.33
Res-TCN	**89.60 ± 0.43**	**92.28 ± 0.32**	**84.10 ± 0.72**	85.01 ± 5.27	88.80 ± 4.51	76.75 ± 7.52
Attention	86.19 ± 0.25	89.65 ± 0.16	79.22 ± 0.69	79.66 ± 4.63	84.88 ± 3.78	68.15 ± 8.46

**Table 3:** Performance metrics of the Cross-subject training and test process over WO dataset considering both the kinematics and the shifted labeling datasets. The highest values for each column are in bold.

Model	WO Dataset—Kinematics Labeling			WO Dataset—Shifted Labeling		
	Accuracy	F Score Stance	F Score Swing	Accuracy	F Score Stance	F Score Swing
Full-BiLSTM	**93.53**	**95.07**	**90.56**	**95.04**	**96.35**	**92.30**
Small BiLSTM	89.74	92.48	83.89	89.87	92.67	83.62
GRU	84.73	88.97	75.22	84.52	89.12	73.18
Res-TCN	88.09	91.28	81.22	87.54	91.19	78.75
Attention	79.57	84.44	70.29	79.61	84.90	68.61

**Table 4:** Performance metrics of the Cross-subject test process over the WT dataset considering both the kinematics and the shifted labeling datasets. The highest values for each column are in bold.

Model	WT Dataset—Kinematics Labeling			WT Dataset—Shifted Labeling		
	Accuracy	F Score Stance	F Score Swing	Accuracy	F Score Stance	F Score Swing
Full-BiLSTM	**92.21**	**94.18**	**88.27**	**92.04**	**94.24**	**87.15**
Small BiLSTM	88.62	91.79	81.45	86.82	90.55	78.23
GRU	85.12	89.31	75.52	83.58	88.57	70.87
Res-TCN	85.67	89.82	75.82	86.63	90.50	77.39
Attention	78.14	83.83	66.25	77.99	83.89	65.27

**Table 5:** Model comparison after the hyperparameters optimization process on WO dataset. *M:* number of units in the second fully connected layer, *P:* number of units in the second BiLSTM layer. * The full-BiLSTM has a different architecture, so it is not possible to directly compare the HP set. The highest *TOS* for each labeling method is reported in bold.

Labeling	Model	Hyperparameter Set [*M P*]	Number of Parameters	*TOS*	Accuracy [%]
Kinematics labeling	Full-BiLSTM	*	1.2 M	0	93.53
	Pruned BiLSTM after HP optimization	[50 100]	994 k	0.35	93.78
	Pruned BiLSTM according to *TOS*	[15 53]	278 k	**0.49**	93.56
Shifted labeling	Full-BiLSTM	*	1.2 M	0	95.04
	Pruned BiLSTM after HP optimization	[27 57]	325 k	**0.37**	94.83
	Pruned BiLSTM according to *TOS*	[27 57]	325 k	**0.37**	94.83

**Table 6:** Results of the computational time analysis.

Model	Time per Window [ms]	Maximum Time [ms]	Memory Usage [MB]	Accuracy [%]
Full-BiLSTM shifted	7.38 ± 1.23	24.71	4.58	95.04
Pruned BiLSTM kinematics	2.72 ± 0.38	4.27	1.06	93.56
Pruned BiLSTM shifted	2.98 ± 0.41	4.21	1.24	94.83
Small BiLSTM shifted	2.01 ± 0.28	3.97	0.70	89.87
GRU shifted	1.81 ± 0.2	3.32	0.21	84.52
Res-TCN shifted	37.84 ± 3.52	50.98	2.93	87.54
Attention shifted	2.61 ± 0.41	3.68	0.57	88.38

## Data Availability

The raw data supporting the conclusions of this article will be made available by the authors upon request.

## Supplementary Materials

The following supporting information can be downloaded at: https://www.mdpi.com/article/10.3390/biomimetics10090617/s1, Figure S1: Full-BiLSTM architecture; Figure S2: Small-BiLSTM architecture; Figure S3: GRU architecture; Figure S4: Res-TCN architecture; Figure S5: Attention architecture; Figure S6: Pruned-BiLSTM architecture.

## Abbreviations

The following abbreviations are used in this manuscript:

DL	Deep Learning
sEMG	Surface Electromyography
EMG	Electromyography
BF	Biceps Femoris
TA	Tibialis Anterior
VL	Vastus Lateralis
SOL	Soleus
COM	Center Of Mass
BiLSTM	Bi-directional Long Short-Term Memory
HP	Hyperparameter/Hyperparameters
GWO	Grey Wolf Optimization
WO	Walking Overground
WT	Walking on a Treadmill
MVIC	Maximal Voluntary Isometric Contraction
IMU	Inertial Measurement Unit
HA	Hip Angle
KA	Knee Angle
RNN	Recurrent Neural Network/Recurrent Neural Networks
GRU	Gated Recurring Unit
Res-TCN	Residual Temporal Convolutional Network
Ad	Accuracy delta
Ac	Accuracy of the candidate model
Ar	Accuracy of the reference model
Pv	Parameters variation
Pc	Parameters of the candidate model
Pr	Parameters of the reference model
Pvc	Parameters variation cost
TOS	Trade-Off Score
ONNX	Open Neural Network Exchange
CV	Cross-validation
LOSO	Leave-One-Subject-Out
CPGs	Central Pattern Generators
