# Supplementary figures and images for: Deep Learning Models Optimization for Gait Phase Identification from EMG Data During Exoskeleton-Assisted Walking
Source: Biomimetics (Basel). 2025 Sep 13;10(9):617. doi: 10.3390/biomimetics10090617 (PMC12467064; doi:10.3390/biomimetics10090617)

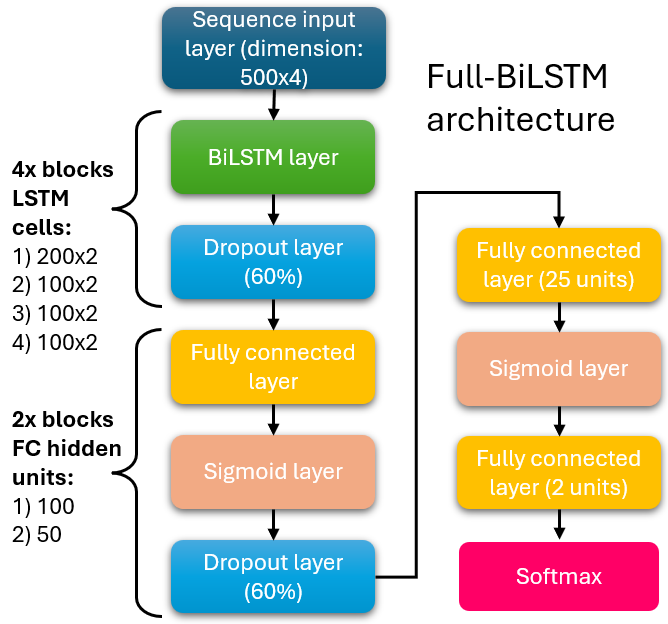

Supplement: Supplementary file 1 [file biomimetics-10-00617-s001.zip › FigureS1_FullBiLSTM_architecture.png]

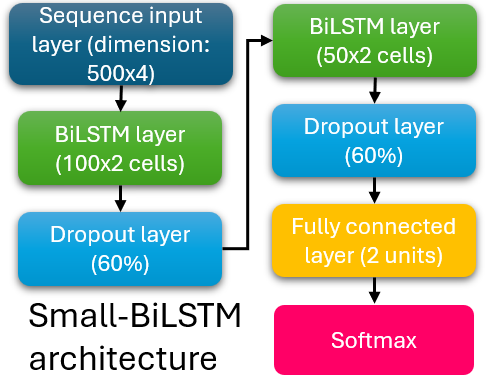

Supplement: Supplementary file 1 [file biomimetics-10-00617-s001.zip › FigureS2_SmallBiLSTM_architecture.png]

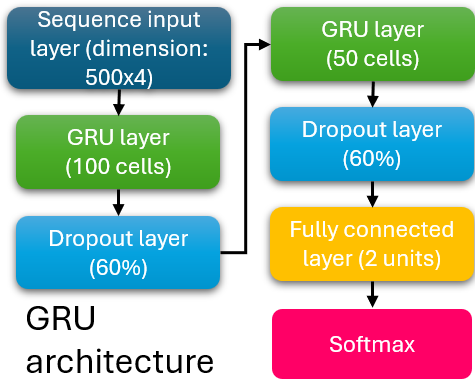

Supplement: Supplementary file 1 [file biomimetics-10-00617-s001.zip › FigureS3_GRU_architecture.png]

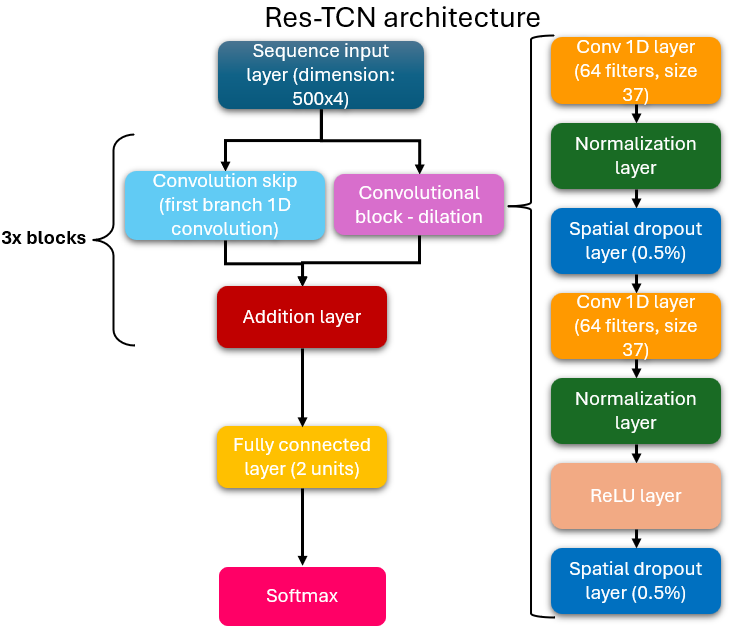

Supplement: Supplementary file 1 [file biomimetics-10-00617-s001.zip › FigureS4_Res-TCN_architecture.png]

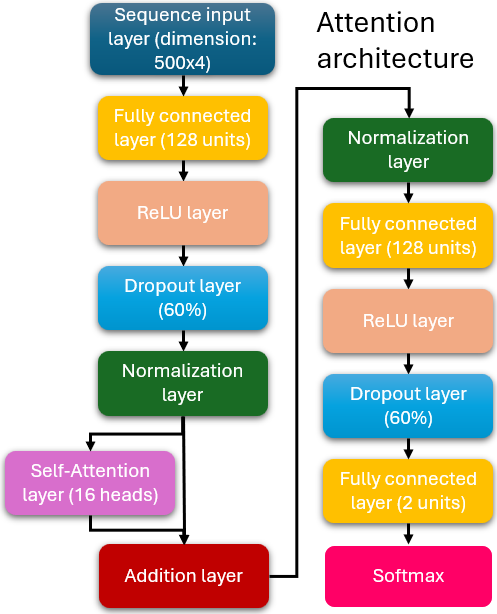

Supplement: Supplementary file 1 [file biomimetics-10-00617-s001.zip › FigureS5_Attention_architecture.png]

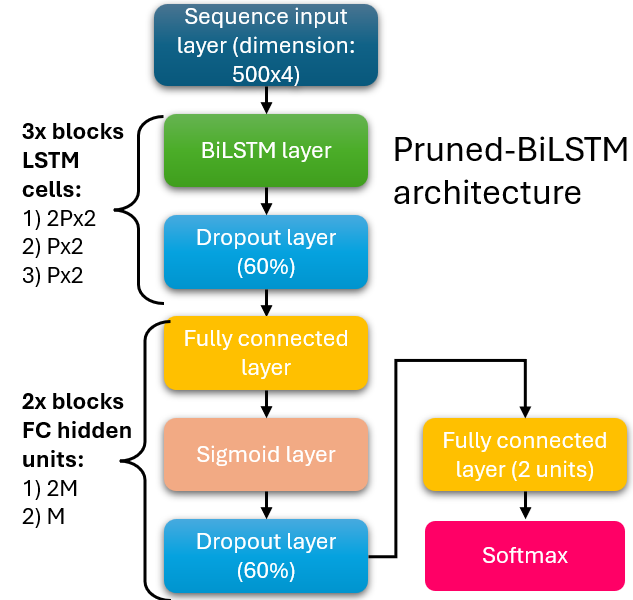

Supplement: Supplementary file 1 [file biomimetics-10-00617-s001.zip › FigureS6_Pruned-BiLSTM.png]
